# The role of quantitative diffusion-weighted imaging in characterization of hypovascular liver lesions: A prospective comparison of intravoxel incoherent motion derived parameters and apparent diffusion coefficient

**DOI:** 10.1371/journal.pone.0247301

**Published:** 2021-02-19

**Authors:** Jelena Djokić Kovač, Marko Daković, Aleksandra Janković, Milica Mitrović, Vladimir Dugalić, Daniel Galun, Aleksandra Đurić-Stefanović, Dragan Mašulović

**Affiliations:** 1 Center for Radiology and Magnetic Resonance Imaging, Clinical Center of Serbia, Belgrade, Serbia; 2 School of Medicine, University of Belgrade, Belgrade, Serbia; 3 Faculty of Physical Chemistry, University of Belgrade, Belgrade, Serbia; 4 First Surgical Clinic, Clinical Center of Serbia, Belgrade, Serbia; Northwestern University Feinberg School of Medicine, UNITED STATES

## Abstract

**Background:**

The utility of intravoxel incoherent motion (IVIM) related parameters in differentiation of hypovascular liver lesions is still unknown.

**Purpose:**

The purpose of this study was to evaluate the value of IVIM related parameters in comparison to apparent diffusion coefficient (ADC) for differentiation among intrahepatic mass-forming cholangiocarcinoma (IMC), and hypovascular liver metastases (HLM).

**Methods:**

Seventy-four prospectively enrolled patients (21 IMC, and 53 HLM) underwent 1.5T magnetic resonance examination with IVIM diffusion-weighted imaging using seven *b* values (0–800 s/mm^2^). Two independent readers performed quantitative analysis of IVIM-related parameters and ADC. Interobserver reliability was tested using a intraclass correlation coefficient. ADC, true diffusion coefficient (*D*), perfusion-related diffusion coefficient (D*), and perfusion fraction (ƒ) were compared among the lesions using Kruskal-Wallis H test. The diagnostic accuracy of each parameter was assessed by receiver operating characteristic (ROC) curve analysis.

**Results:**

The interobserver agreement was good for ADC (0.802), and excellent for *D*, D*, and ƒ (0.911, 0.927, and 0.942, respectively). ADC, and *D* values were significantly different among IMC and HLM (both *p* < 0.05), while there was no significant difference among these lesions for ƒ and D* (*p* = 0.101, and *p* = 0.612, respectively). ROC analysis showed higher diagnostic performance of *D* in comparison to ADC (AUC = 0.879 vs 0.821).

**Conclusion:**

IVIM-derived parameters in particular *D*, in addition to ADC, could help in differentiation between most common hypovascular malignant liver lesions, intrahepatic mass—forming cholangiocarcinoma and hypovascular liver metastases.

## Introduction

Diffusion-weighted imaging (DWI) is increasingly used in everyday clinical magnetic resonance imaging (MRI) for focal liver lesions detection, characterization, and assessment of treatment response. It provides information on the cellular density without the need for intravenous contrast administration [1]. Besides visual assessment, DWI enables quantitative evaluation in the form of apparent diffusion coefficient (ADC) measurements [2]. Although many previous studies reported the utility of DWI in differentiation among malignant and benign liver lesions, the great overlap of ADC values limits its use in clinical practice [3,4]. Knowing that ADC is compound parameter affected by both blood flow and molecular diffusion can partly explain these results [5]. In order to overcome the drawbacks of ADC, the intravoxel incoherent motion (IVIM) model which relies on the use of multiple *b*-values was introduced by Le Bihan in 1986 [6]. Using multiple *b* values according to a bi-exponential model, IVIM imaging enables separation and evaluation of contributions of perfusion and true molecular diffusion in the form of *D* (true diffusion coefficient), D* (perfusion-related diffusion coefficient), and ƒ (perfusion fraction) [7,8]. Regarding the use of IVIM in the evaluation of focal liver lesions, previous studies mainly focused on differentiation of malignant from benign lesions [5,9–12]. However, most of these studies included both solid and non-solid tumors, such as hemangiomas and cysts [9–12]. Two studies aimed to make distinction among hypervascular benign and malignant lesions using IVIM-DWI [13,14]. Nevertheless, there are no data in the previous literature concerning the value of IVIM-derived parameters in the differentiation of hypovascular liver lesions.

The most common malignant hypovascular liver lesions are liver metastases and intrahepatic cholangiocarcinoma. According to the growth pattern, intrahepatic cholangiocarcinoma can be classified into three subtypes, with mass-forming type being the most frequent [15,16]. In a daily clinical work the distinction between intrahepatic mass-forming cholangiocarcinoma (IMC) and hypovascular liver metastases (HLM) is usually not difficult, since the primary origin of the tumor in a case of metastases is often already known [17]. Furthermore, ancillary features like capsular retraction, and peripheral biliary dilatation favor the diagnosis of IMC [18]. Nevertheless, a small subset of patients present with a metastatic disease of unknown primary [19]. Additionally, the patients with known malignant disease and liver metastases could develop IMC independently of their primary disease. Since literature data about the usefulness of IVIM-DWI in the differentiation of hypovascular liver lesions are very scarce, the purpose of this study was to determine the value of IVIM-related parameters in comparison to ADC for their characterization.

## Materials and methods

### Patient characteristics

From January 2017 to December 2019, a total of 208 consecutive patients who underwent MRI including IVIM were enrolled in this prospective study. Primary inclusion criterion was clinical suspicion of solid focal liver lesion based on findings of previous examinations. The exclusion criteria were: 1) no liver lesion on MRI; 2) MRI diagnosis of liver cysts and hemangiomas; 3) patients with hypervascular liver lesions; 3) the lesion diameter less than 2 cm; 4) patients who had no history of previous chemotherapy treatment; 5) patients whose image quality was reduced due to the presence of motion or misregistration artifacts; 6) patients with no pathohistological proof of malignant hypovascular liver lesions. Finally, a total of seventy-four patients with histologically proven IMC (n = 21; mean size: 5.3±2.1 cm; mean age: 68±11 years), and HLM (n = 53; mean size: 3.8±1.9 cm; mean age: 59±12 years) were included in the study. In order to avoid clustering effect, only the largest lesion was included in the analysis in patients with multiple tumors. The primary tumor sites in patients with liver metastases were as follows: colorectal cancer (n = 42); pancreatic cancer (n = 7); gastric cancer (n = 2); and Vater ampulla cancer (n = 2). IMCs were diagnosed after surgical resection in 13 patients, and after liver biopsy in eight patients. The diagnosis of metastases was confirmed after surgical resection in 37 patients, and after laparoscopic or percutaneous biopsy in 16 patients. The study was approved by an Ethical Comittee of School of Medicine, University of Belgrade, and a written informed consent was obtained from all participants.

### MRI examination

#### Image acquisition

All patients underwent MRI prior to pathologic diagnosis (mean interval time 15 days; range 1–32 days) at 1.5 T (Signa HDxt, GE Healthcare, Waukesha, Wisconsin, USA). All images were obtained using an 8-channel phased-array abdominal coil and spine array coil to optimize signal-to-noise ratio. A rectangular field of view of 320 to 400 mm was adjusted for each patient’s body size and was held constant for all sequences. Routine breath hold gradient recalled echo (GRE) (in- and out-of-phase) T1-weighted sequence, a breath-hold T2-weighted single-shot fast spin echo sequence, a breath-hold T2-weighted fat-suppressed (FS), as well as breath-hold T1-weighted FS GRE sequence were performed. Dynamic imaging was performed after a rapid bolus of a standard dose of 0.1 mmol/kg gadopentetate dimeglumine (*n* = 57), and a 0.025 mmol/kg of gadoxetic acid (*n* = 17). Contrast-enhanced images were acquired with 3D-GRE sequences in late hepatic arterial phase (24 s), portal venous phase (1 min) and interstitial phase (2 min) in the axial plane for all phases, and coronal plane only for interstitial phase. Hepatobiliary phase images were acquired at 20 minutes after injection. The parameters of each pulse sequence are summarized in Table 1.

**Table 1:** Parameters of sequences used at 1.5T MRI scanner.

Parameter	T2-weighted single shot FSE	T2-weighted FS	T1-weighted in-phase	T1-weighted out-of phase	DW SSSE EPI	T1-weighted 3D-GRE
TR (ms)	1200	1200	160	160	5000	6,7
TE (ms)	90	90	4.6	2.328	52	4,3
Flip angle	90	90	80	80	180	15
BW/pixel (Hz)	244.141	244.141	244.141	244.141	1953.12	83.33
Matrix (phase x freuequency)	224x288	224x288	192x256	192x256	136x136	192x320
FOV (cm)	40	40	40	40	36	40
Section thickness (mm)	5	5	5	5	7	4.4
Intersectional gap (%)	20	20	20	20	0	50
No. of signal acquisition	4	1	1	1	3	1
Fat suppresion	None	Fat sat	None	None	None	Fat sat
Respiratory control	BH	BH	BH	BH	RT	BH

TR, repetition time; TE, echo time; Hz, Hertz; FOV, field of view; FSE, fast spin echo; GRE, gradient recalled echo; FS, fat supressed; 3D, three dimensional; DW, diffusion weighted; SSSE, single shot spin-echo; EPI, echo planar imaging; BW, bandwidth; BH, breath hold; RT, respiratory triggered.

All DWI examinations were obtained with a respiratory triggered single-shot spin echo-planar imaging with multiple *b* values (10, 25, 50, 100, 200, 400, and 800 s/mm^2^) in three orthogonal gradient directions. DWI was performed before intravenous injection of contrast media. In cases where gadoxectic acid was administered, DWI was performed before hepatobiliary phase. The imaging parameters are presented in Table 1. The acquisition was separated in blocks (*b*0, *b*10), (*b*0, *b*25), (*b*0, *b*50)…(*b*0, *b*800), each acquired in a single breath-hold in expiration (TA = 24 s) to avoid motion artefacts.

#### Processing of DWI images and determination of IVIM parameters

The diffusion weighted images were analyzed by two abdominal radiologists (11 years and 5 years of experience) who were blinded to clinical and pathological data. T2-weighted images and dynamic contrast-enhanced T1-weighted images were used to improve lesion localization and to avoid areas of necrosis. Regions of interest (ROI) were manually positioned carefully at least 1mm away from the margin of the tumor, on diffusion-weighted images for *b*0, and then automatically copied to all parametric maps. If the lesion was homogeneous, three circular ROIs were placed on contiguous slices which covered the largest portion of the lesion, encompassing as much of the lesion as possible avoiding vessels and areas of necrosis. If a lesion demonstrated heterogeneous appearance, three circular ROI of equal radii were drawn on viable parts of the tumor, defined as region with maximal contrast enhancement.

The data for ƒ, *D* and D* were obtained by fitting signal values from manually positioned ROIs. Model fitting was implemented in statistical package R [20]. The data were fitted using nonlinear least square (nls) method to equation.

$$\text{S}(b)/{\text{S}}_{0}=ƒ\;\text{x}\;\text{exp}\;(-b\;\text{x}\;D*)+(1-\text{f}\;)\;\text{exp}(-b\;\text{x}\;D)$$ (1)

where S(*b*) corresponds to mean signal intensity on DWI under the given *b* value. S_0_ is the mean signal intensity on DWI with *b* value = 0. D* is perfusion-related diffusion parameter representing incoherent circulation, *D* represents pure molecular diffusion, and ƒ is the fraction of the diffusion linked to the microcirculation. This method was chosen because the nls algorithm estimates errors of the obtained parameters.

Quantitative ADC maps were calculated on voxel-by-voxel basis using commercial workstation for combination of *b* = 0 and *b*_*1*_ = *800* using the equation:

$$\text{ln}({\text{S}}_{1}/{\text{S}}_{0})=-b_{1}\text{ADC}$$ (2)

where S_0_ and S_1_ correspond to signal intensities for *b* values 0 and *b* = 800 s/mm^2^.

The final values of ƒ, *D*, D* and ADC for *b* = 800 s/mm^2^ were calculated by averaging the three measurements. In addition, just for illustration purposes, we generated the maps of ƒ, *D* and D* parameters using MITK Diffusion software [21] for two patients. In order to achieve this, the sotware uses the Eq 1 to fit signal values in each voxel.

### Statistical analysis

For the assessment of normal distribution of statistical data, Kolmogorov-Smirnov test was used. Continuous variables were presented as mean values ± standard deviation (or median in cases of non normal distribution). Interobserver reliability of the measurements between two readers was assessed by using intraclass correlation coefficient (ICC): ICC values less than 0.5 indicated poor reliability; values between 0.5 and 0.75 indicated moderate to good reliability; and values higher than 0.9 indicated excellent reliability. To determine whether there was a significant difference between ADC, *D*, D*, and ƒ among the groups (IMC, and HLM) the Kruskal-Wallis H test was used followed by Steel-Dwass post hoc test. Parameters with statistical significance among two groups were evaluated by a receiver operating characteristic (ROC) analysis in order to determine the diagnostic accuracy. Cut-off values corresponding to the best sensitivity and specificity were determined using Youden test. The areas under the ROC curve (AUC) were compared to assess the differences between parameters. Statistical significance level was set at 0.05. All analyses were performed with software SPSS (version 17.0 for Windows; SPSS, Chicago, Ill).

## Results

### Reliability of ADC and IVIM-derived parameters

The interobserver agreement of the measurements for liver lesions was moderate to good for ADC and excellent for IVIM-derived parameters, with ICC values of 0.802 for ADC maps, 0.911 for *D*, 0.927 for D*, and 0.942 for ƒ.

### ADC and IVIM-derived parameters of hypovascular liver lesions

**Fig 1:**
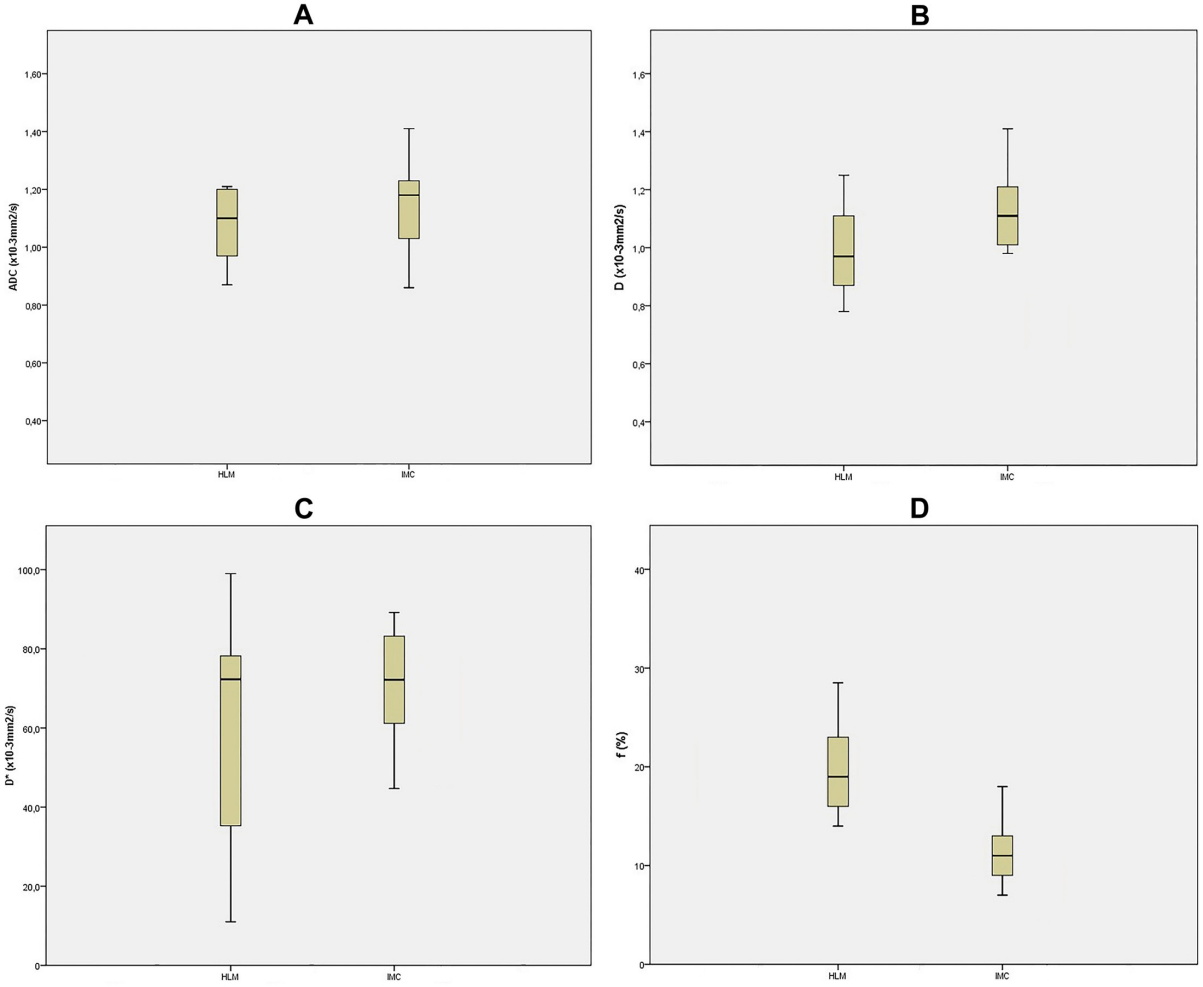
Box plots of ADC and IVIM-derived parameters. Box plot of ADC (A), *D* (B), D* (C), and ƒ (D) for intrahepatic mass-forming cholangiocarcinoma (IMC), and hypovascular liver metastases (HLM), The middle line represents the median. The central box represents the measurements from the lower to the upper quartile. Error bars indicate the range from the maximum to the minimum parameters measurement. ADC, apparent diffusion coefficient; *D*; true diffusion coefficient; D* perfusion-related diffusion coefficient; ƒ, perfusion fraction.

Mean values of ADC and IVIM-derived parameters for different types of hypovascular liver lesions are shown in Table 2, while corresponding box plots are illustrated in Fig 1.

**Table 2:** Mean values of ADCs and IVIM-related parameters.

Parameter	HLM (n = 53)	IMC (n = 21)	*p* value*
ADC (·10^−3^ mm^2^/s)	1.09±0.03	1.22±0.21	< 0.05
*D* (·10^−3^ mm^2^/s)	0.92±0.13	1.13±0.21	< 0.05
D* (·10^−3^ mm^2^/s)	36.18±13.41	19.11±5.28	0.11
ƒ (%)	17.51±2.23	11.21±2.31	0.08

Data are expressed as means ± standard deviation. IMC-intrahepatic mass-forming cholangiocarcinoma; HLM-hypovascular liver metastases; ADC-apparent diffusion coefficient; *D*-true diffusion coefficient; D*- perfusion-related diffusion coefficient; ƒ-perfusion fraction.

*Kruskal-Wallis test.

**Fig 2:**
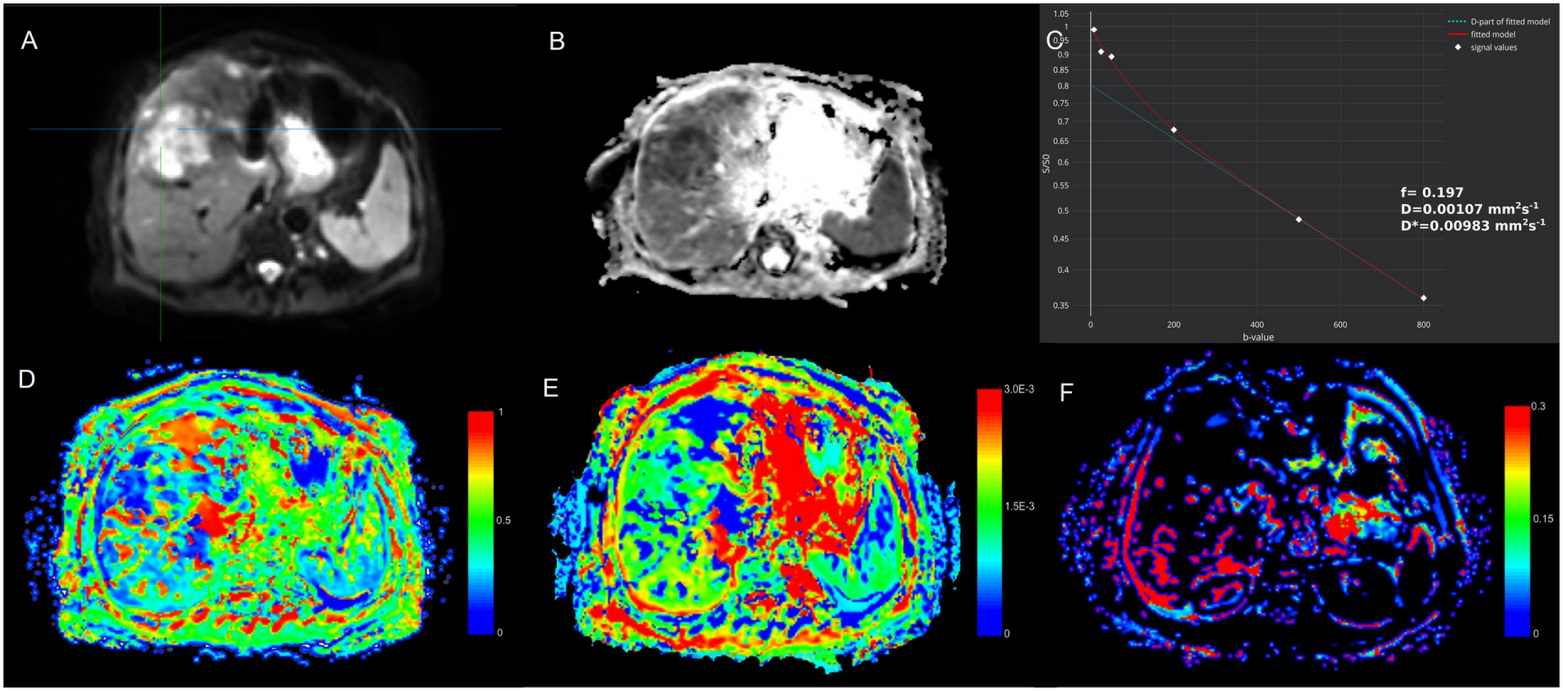
The illustration of IVIM-DWI analysis (MITK Diffusion software [21]) in 63-year old woman with 5.1 cm surgically confirmed intrahepatic mass-forming cholangiocarcinoma. The tumor is heterogeneously hyperintense on diffusion weighted image with *b* = 0 s/mm^2^ (**A**). ADC map (*b* = 800 s/mm^2^) is shown on **B**, and signal attenuation curve on **C**. Parametric maps for *D* (**D**), D* (**E**), and ƒ (**F**). The mean ADC, *D*, D*, and ƒ values of the lesion were 1.19±0.06·10^−3^ mm^2^/s, 1.09±0.03·10^−3^ mm^2^/s, 18.81±9.31·10^−3^ mm^2^/s, 15.08±2.81%, resepectively. The mean ADC, *D*, D*, and ƒ values of the healthy liver parenchyma were 1.51±0.07·10^−3^ mm^2^/s, 1.42±0.09·10^−3^ mm^2^/s, 34.51±10.17·10^−3^ mm^2^/s, 23.11±8.32%, resepectively. ADC, apparent diffusion coefficient; *D*, true diffusion coefficient; D*, perfusion-related diffusion coefficient; ƒ, perfusion fraction.

**Fig 3:**
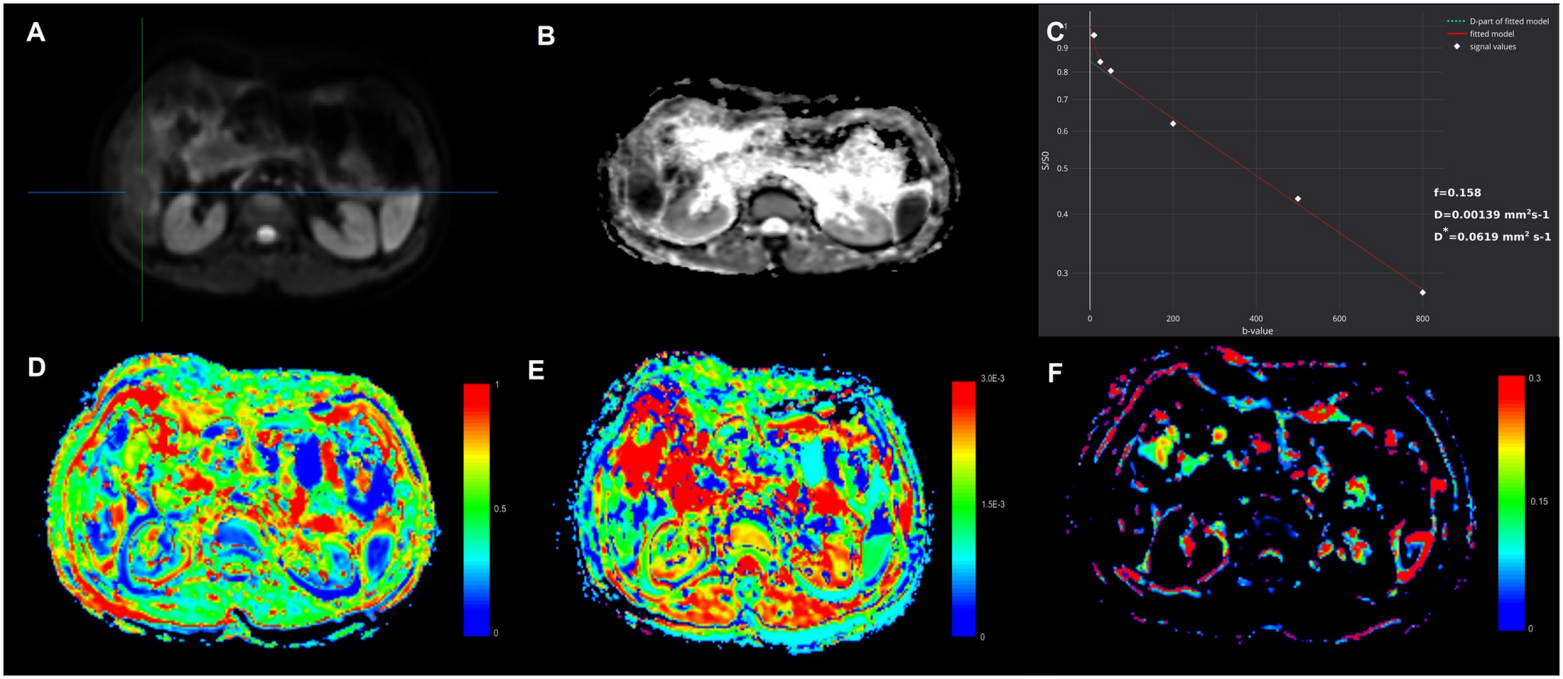
The illustration of IVIM-DWI analysis (MITK Diffusion software [21]) in 51-year old man with 3.2 cm surgically confirmed liver metastasis in liver segment VI from rectal cancer. Diffusion weighted image with *b* = 0 s/mm^2^ shows tumor as hyperintense mass (**A**). ADC map (*b* = 800 s/mm^2^) is shown on **B**, and signal attenuation curve on **C**. Parametric maps for *D* (**D**), D* (**E**), and ƒ (**F**). The mean ADC, *D*, D*, and ƒ values of the lesion were 1.11±0.04·10^−3^ mm^2^/s, 0.99±0.06·10^−3^ mm^2^/s, 39.53±11.22·10^−3^ mm^2^/s, 19.81±3.21%, resepectively. The mean ADC, *D*, D*, and ƒ values of the healthy liver parenchyma were 1.47±0.09·10^−3^ mm^2^/s, 1.35±0.06·10^−3^ mm^2^/s, 29±12.01·10^−3^ mm^2^/s, 21.08±7.61%, resepectively. ADC, apparent diffusion coefficient; *D*, true diffusion coefficient; D*, perfusion-related diffusion coefficient; ƒ, perfusion fraction.

Analysis of multiple comparisons between groups showed significant differences for ADC, and *D* between different lesions (Table 3). While D*, and ƒ were not significantly different between HLM and IMC (*p* = 0.612, and 0.101, respectively), there were significant differences in *D* and ADC (both *p* < 0.05; Figs 2 and 3).

**Table 3:** Steel-Dwass test results of multiple comparisons.

	ADC (·10^−3^ mm^2^/s)	*D* (·10^−3^ mm^2^/s)	D* (·10^−3^ mm^2^/s)	ƒ (%)
**IMC vs HLM**	0.021	0.019	0.612	0.101

Data are *p* values.

IMC-intrahepatic mass-forming cholangiocarcinoma; HLM-hypovascular liver metastases; ADC-apparent diffusion coefficient; *D*-true diffusion coefficient; D*- perfusion-related diffusion coefficient; ƒ-perfusion fraction.

### Diagnostic performance of ADC and IVIM-derived parameters

**Fig 4:**
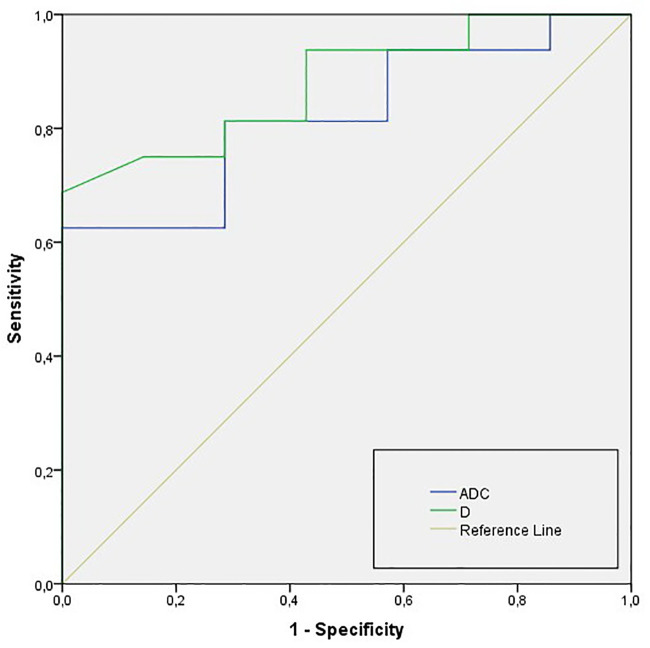
ROC curves of ADC and *D* for differentiation of IMC from HLM IMC-intrahepatic mass-forming cholangiocarcinoma; HLM-hypovascular liver metastases; ADC, apparent diffusion coefficient; *D*, true diffusion coefficient.

The diagnostic performance of ADC and IVIM-related parameters is summarized in Table 4. In particular, *D* had higher AUC for differentiation of IMC from HLM in comparison to ADC (AUC = 0.879, and 0.821, respectively). Nevertheless, no significant differences were found between *D* and ADC using pairwise comparison of ROC curves (*p* = 0.412; Fig 4).

**Table 4:** Results of ROC analysis for differentiation of hypovascular liver lesions.

Comparison	AUC †	Cut off value	Sensitivity (%)	Specificity (%)
**IMC vs HLM**				
ADC (·10^−3^ mm^2^/s)	0.821 (0.721–0.932)	1.10	78.9	82.8
*D* (·10^−3^ mm^2^/s)	0.879 (0.801–0.916)	0.99	92.2	91.0

ROC- receiver operating curve; AUC-area under the curve; IMC-intrahepatic mass-forming cholangiocarcinoma; HLM-hypovascular liver metastases; ADC-apparent diffusion coefficient; *D*-true diffusion coefficient.

^†^ Numbers in parentheses are 95% confidence interval.

## Discussion

The results from the current study have shown that *D* and ADC had the highest diagnostic accuracy for differentiation between two most common malignant hypovascular liver lesions, IMC and HLM. These results could be explained by different pathological characteristics of these lesions. While HLM are highly cellular tumors with small amount of extracellular space resulting in very restricted diffusion, IMC is characterized by large interstitial space and low cellularity [22]. Although both *D* and ADC were significantly higher in IMC compared to HLM, *D* slightly outperformed ADC (AUC values of 0.879 and 0.821, respectively). To our knowledge there are no data in the previous literature concerning the value of IVIM-derived parameters for the differentiation of IMC from HLM. Several prior studies reported analysis of IVIM-related parameters for distinction of benign from malignant liver lesions which included IMC and HLM [9–12]. While Zhu et al. [5] did not show any advantage of *D* compared to ADC, *D* was found to be the most reliable predictor of malignancy in the study by Yoon et al. [9], and Choi et al. [23]. The superiority of *D* which was also found in our study could be explained by the fact that ADC is compound factor containing both diffusion and perfusion components [6]. Since malignant liver lesions can have at the same time diffusion restriction and increased perfusion, these two effects may cancel each other thus making ADC a less sensitive biomarker [24]. Opposite to ADC, *D* represents pure diffusion parameter [6].

Regarding our absolute mean *D* values they were in line with previous reports with 1.13±0.21·10^−3^ mm^2^/s for IMC and 0.92±0.13·10^−3^ mm^2^/s for HLM in the present study compared to 1.19±0.35·10^−3^ mm^2^/s and 0.93±0.36·10^−3^ mm^2^/s in the study by Wang et al. [25]. On the other hand, Choi et al. [23] reported 1.31±0.07·10^−3^ mm^2^/s for IMC and 1.10±0.19·10^−3^ mm^2^/s for HLM. Nevertheless, previous reports included metastases from both hypervascular and hypovascular primary tumors, thus precluding the precise comparison between studies. Concerning ADC values of IMC the results from literature are not consistent [25,26]. Although slightly lower, the ADC values (1.22±0.21·10^−3^ mm^2^/s) in the current study are comparable to those of Doblas et al. [12] (1.31±0.21·10^−3^ mm^2^/s), while Fattach et al. [27] found a mean ADC of 1.042·10^−3^ mm^2^/s. These discrepancies might be the consequence of different diffusion sensitivity coefficient used throughout the studies [4]. A second reason could be attributable to the difference in the method of ROI placement. Namely, IMC are known to be heterogeneous lesions with loose fibrotic stroma in central parts and densely packed tumor cells at the periphery. In this regard, it was shown that if ROI was placed on the periphery of the lesion, ADC values were significantly lower compared to ROI encompassing whole lesion (0.813±0.221·10^−3^ mm^2^/s vs 1.001±0.112·10^−3^ mm^2^/s) [28]. In the present study ROI included both the periphery and center of the lesion which could explain slightly higher values than those previously reported. The importance of the method of ROI placement was also stressed in the study by Wei et al. [29].

The value of perfusion related IVIM parameters in the evaluation of focal liver lesions has been extensively studied previously. Yoon et al. [9] found that D* and ƒ were significantly different between hypervascular and hypovascular liver lesions, indicating that using these parameters the vascularity of the lesion could be assessed even without contrast administration. In this regard, the significant positive correlation was found between ƒ and relative blood volumes, while pseudodiffusion coefficient was correlated with relative blood flow [30]. However, to date no added value of perfusion related IVIM parameters was found in differentiation between benign and malignant lesions [9–12]. The reason why no differences in D* and ƒ were observed is unclear but may be because blood volume, blood flow, or secretion have different effects on perfusion properties in different lesion types [30]. Moreover, the main difference between benign and malignant lesion is in their cellularity while microvessel density does not have to be different [13]. For ƒ and D* we did not find significant differences between IMC and HLM, although ƒ values of IMC showed a trend toward lower values (11.21±2.31%, and 17.51±2.23%, respectively). Since both lesions are hypovascular, these data are in accordance with their widely known contrast-enhancement behaviour on computed tomography and MRI [15,17]. Similarly to prior studies which have demonstrated that error in calculation of D* is high despite standardized measurement, in the present study D* values showed high standard deviation [31]. Also, the reported coefficient of reproducibility for D* measurement was as high as 2100% in the study by Lemke et al. [32]. These results indicate that D* can not be used as reliable quantitative parameter for perfusion analysis. Our absolute mean values of ƒ for IMC, and HLM were similar to previously published results of Choi et al. [23]. Nevertheless, ƒ values were found to be much higher in the study by Doblas et al. [12]. The differences in ƒ values could partly be explained by different number of *b* values which varied among studies. While Luo et al. [33] used eleven *b* values, 8 to 12 *b* values are used in other reports [9–14,23], which is in line with the present study. Besides overall number of used *b* values, the number of small *b* values (<200 s/mm^2^) is very important for the accurate measurement of perfusion parameters [7].

In the current study, we showed good interobserver reliability between ADC and IVIM-related parameters. Similar results were previously reported by Klauss et al. [13] with correlation coefficients of 0.81, 0.81, 0.84, and 0.58 for ƒ, D, D* and ADC, respectively. Consistently, in the recent study evaluating the value of IVIM-DWI of solid pancreatic masses the interobserver agreement was excellent for IVIM-related parameters ranging from ICC values of 0.860 for ADC up to 0.983 for ƒ [34].

Our study has several limitations. First, we included only lesions larger than 2 cm in order to minimize measurement error from the partial volume averaging effect. Second, the method for ROI placement might have influenced ADC and IVIM-derived parameters measurements. In particular, we put three circular ROI in viable tumor tissue avoiding areas of necrosis, leading to nonuniform ROI placement in all lesions. However, no optimal method for the ROI placement in heterogeneous tumors has yet been determined. In addition, we evaluated only interobserver reliability, but did not evaluate reproducibility of the method.

In conclusion, our study shows that IVIM-derived parameters, in particular *D*, in addition to ADC are helpfull non-invasive diagnostic modality for differentiation of the most common hypovascular malignant liver lesions, intrahepatic mass-forming cholangiocarcinoma and hypovascular liver metastases.
